# Association of Hematocrit and Albumin Difference With Ventilator-Associated Pneumonia in Patients With Continuous Mechanical Ventilation: Evidence From MIMIC-IV Database

**DOI:** 10.1155/carj/6084081

**Published:** 2025-11-06

**Authors:** Weiwei Mao, Chengyun Mu

**Affiliations:** Department of Emergency ICU, Lianyungang Hospital of Traditional Chinese Medicine, Lianyungang Affiliated Hospital of Nanjing University of Chinese Medicine, Lianyungang 222004, Jiangsu, China

**Keywords:** difference of hematocrit and albumin, mechanical ventilation, subgroups, ventilator-associated pneumonia

## Abstract

**Objective:**

This study aims to investigate the relationship between the difference in hematocrit and albumin (HCT-ALB) and ventilator-associated pneumonia (VAP) among patients undergoing continuous mechanical ventilation.

**Methods:**

This research utilized the data from the Medical Information Mart for Intensive Care IV database. The primary outcome was VAP occurred. HCT-ALB levels were divided into three groups according to the quantile: < −1.10; −1.10–5.30; ≥ 5.30. All patients with continuous mechanical ventilation were categorized into two groups: those who developed VAP and those who did not. Univariate and multivariate logistic regression analyses were used to assess the relationship between HCT-ALB and VAP risk. Receiver operating characteristic (ROC) curves were used to evaluate the predictive ability. To further assess the robustness of the findings, subgroup analyses were performed.

**Results:**

In our study, a total of 3021 patients were enrolled, and among them, 361 patients experienced VAP. Multivariate logistic regression showed that taking HCT-ALB < −1.10 as reference, HCT-ALB ≥ 5.30 was linked to an increased risk of VAP in patients undergoing continuous mechanical ventilation (odds ratio = 1.36, 95% confidence interval: 1.02–1.81). The ROC curve demonstrated approximately moderate predictive ability. This association remained robust in subgroups of male, quick Sepsis-Related Organ Failure Assessment score ≤ 2, not using antibiotics, having oral care, and no history of trauma injury, chronic obstructive pulmonary disease, or respiratory failure.

**Conclusion:**

HCT-ALB, as an easily measurable indicator, was associated with the risk of VAP in patients with continuous mechanical ventilation.

## 1. Introduction

Mechanical ventilation, a widely employed method for assisted ventilation in the intensive care unit (ICU), accounts for over 25% of critically ill patients and is associated with diverse complications as well as a heightened risk of mortality [1]. Ventilator-associated pneumonia (VAP) is one of the most common infections that occur in the ICU, frequently observed in patients receiving continuous mechanical ventilation for over 48 h [2]. Among critically ill patients receiving continuous mechanical ventilation, those with VAP showed a higher risk of mortality compared to those who did not have VAP [3]. Therefore, identifying high-risk patients who are prone to developing VAP holds significant clinical implications in terms of reducing the burden on the ICU.

Albumin (ALB) is a negative acute-phase protein, serving as a crucial indicator for assessing nutrition and inflammation status [4]. Hypoalbuminemia is prevalent among critically ill patients and frequently leads to an unfavorable prognosis [5]. It is reported that hypoalbuminemia was linked with the acquisition and severity of infectious diseases [6]. Hematocrit (HCT) represents the proportion of red blood cells to the total volume of blood, and it plays a crucial role in evaluating the prognosis of critically ill patients and serves as an important indicator for fluid resuscitation therapy [7]. Recently, the difference between HCT and ALB (HCT-ALB) has been suggested as a potential biomarker for infectious diseases [8–10]. A case-control study demonstrated that patients with infectious diseases exhibit significantly elevated levels of HCT-ALB compared to those without infectious diseases. Moreover, HCT-ALB exhibits substantial diagnostic value in distinguishing severe infections and can function as a reliable biomarker for diagnosing infectious diseases [11]. In addition, elevated levels of HCT-ALB in ICU patients were significantly linked to a greater mortality risk among elderly individuals with sepsis [12]. However, the association of HCT-ALB with VAP risk in mechanically ventilated patients remains unclear.

This study intended to examine the association of HCT-ALB with VAP risk in patients undergoing continuous mechanical ventilation, which can provide evidence for clinical decision-making.

## 2. Materials and Methods

### 2.1. Study Population

The data utilized in this retrospective cohort study were obtained from Medical Information Mart for Intensive Care IV v2.2 (MIMIC-IV v2.2). The data were extracted in November 2023. MIMIC-IV is a large, free, and open database that contains comprehensive information on all patients admitted to the ICUs at Beth Israel Deaconess Medical Center (BIDMC) between 2008 and 2019 [13]. Comprehensive patient information, including vital signs, laboratory results, medications, procedures, and length of hospital stay, was documented. All personal data are deidentified through the use of a random code to replace patient identification, and this study was approved by the Institutional Review Board (IRB) of BIDMC with a waiver of informed consent.

**Figure 1:**
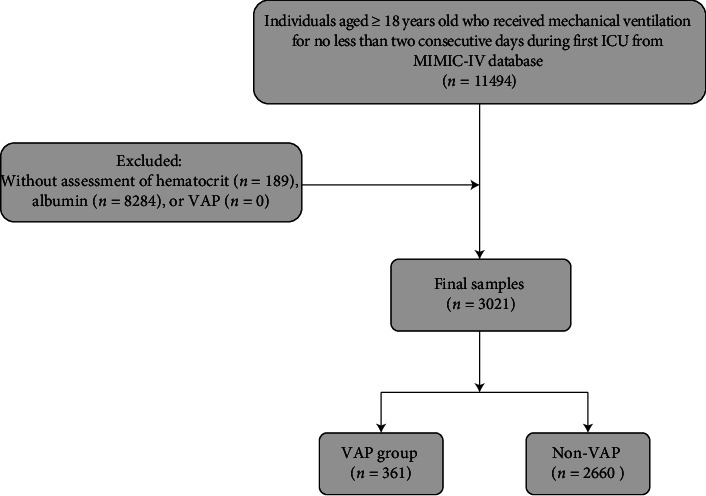
Flowchart of this study.

As shown in Figure 1, we included individuals aged ≥ 18 years old who received mechanical ventilation for no less than two consecutive days during their first ICU admission (*n* = 11,494). Individuals without assessment of HCT, ALB, or VAP were excluded. Ultimately, a study cohort of 3021 patients was established and categorized into two groups based on whether or not VAP occurred.

### 2.2. Outcomes and Confounders

The primary outcome was whether VAP occurred. VAP was defined according to the International Classification of Diseases 9th edition (ICD-9) codes 4957 and 99731, as well as the International Classification of Diseases 10th edition (ICD-10) code J95851. The HCT-ALB index was calculated as HCT (%) minus ALB (g/L). HCT-ALB levels were divided into three groups according to the quantile: < −1.10; −1.10–5.30; ≥ 5.30.

Potential confounders extracted included age (years), gender, race/ethnicity, insurance status, and ICU type, mean arterial pressure (MAP, mmHg), heart rate (bpm), temperature (°C), white blood cell (WBC, K/L), platelet (K/L), hemoglobin (g/dL), creatinine (mg/dL), international normalized ratio (INR, %), prothrombin time (PT, seconds), glucose (mg/dL), blood urea nitrogen (BUN, mg/dL), sodium (mEq/L), chloride (mEq/L), bicarbonate (mEq/L), oxygen saturation (SpO_2_, %), quick Sepsis-related Organ Failure Assessment (qSOFA), trauma injury, antibiotics, oral care, diabetes, chronic obstructive pulmonary disease (COPD), and respiratory failure. ICU types are categorized as Neurological, Cardiology, Medical, Surgical, Medical/Surgical, and other ICUs. In this study, respiratory failure refers to chronic respiratory failure diagnosed before the start of mechanical ventilation. All clinical variables and laboratory data were taken from the first record within 24 h after mechanical ventilation initiation.

### 2.3. Statistical Analysis

All statistical analyses were performed using SAS Version 9.4. A *p* value threshold of less than 0.05 was used to denote a statistically significant difference. Continuous variables were reported as mean and standard deviation (Mean [±SD]) or median and interquartile spacing (M [Q_1_, Q_3_]), while categorical variables were presented as the number of cases and composition ratio (*n* [%]). For comparing continuous variables across different groups, *t*-tests or Wilcoxon rank-sum tests were utilized. Categorical variables across the groups were analyzed using the Chi-squared test. The missing values of variables were imputed using the predictive mean matching method, and a sensitivity analysis was performed both before and after the data interpolation (Supporting Table 1). A univariate logistic regression model was constructed to determine confounders related to VAP. Subsequently, both univariate and multivariate logistic regression models were employed to assess the relationship of HCT-ALB with VAP risk among patients with continuous mechanical ventilation. Model 1: univariate logistic regression; Model 2: multivariate logistic regression, adjusting for all potential confounders. The odds ratio (OR) along with 95% confidence interval (CI) was calculated.

A restricted cubic spline plot was used to explore the nonlinear relationship of HCT-ALB and the risk of VAP. Furthermore, to assess the predictive ability and accuracy of the adjusted model, we employed receiver operating characteristic (ROC) curves, area under the curve (AUC), and calibration curves. To further test the stability of results, subgroup analyses were conducted in gender (male/female), qSOFA score (> 2/≤ 2), trauma injury (No/Yes), antibiotics (No/Yes), oral care (No/Yes), COPD (No/Yes), and respiratory failure (No/Yes) subgroups.

## 3. Results

### 3.1. Characteristics of all Subjects

Table 1 presents the baseline characteristics of 3021 patients, with a mean (±SD) age of 61.73 ± 16.54 years. The numbers of females and males were 1259 (41.67%) and 1762 (58.33%), respectively. Simultaneously, the whole cohort is divided into two groups: VAP (*n* = 361) and non-VAP (*n* = 2660). Baseline characteristics were compared between the groups. Statistically significant differences were observed between the two groups in terms of age, race/ethnicity, insurance status, sodium, chloride, qSOFA, trauma injury, and respiratory failure (*p* < 0.05). Compared to non-VAP patients, participants with VAP had a higher sodium, chloride, qSOFA score, and incidence of trauma injury and respiratory failure.

### 3.2. Relationship Between HCT-ALB and the Risk of VAP

Supporting Table 2 indicates the possible confounders related to VAP risk, including age, race/ethnicity, insurance status, sodium, chloride, qSOFA, trauma injury, and respiratory failure. As illustrated in Table 2, the univariate logistic regression model revealed that the high HCT-ALB group (≥ 5.30) exhibited an elevated risk of VAP compared to the reference group (HCT-ALB < −1.10) (Model 1: OR = 1.50, 95% CI: 1.14–1.98, *p*=0.004). After accounting for all confounding factors, we found that high HCT-ALB (≥ 5.30) remained linked to a higher risk of VAP in patients with continuous mechanical ventilation (Model 2: OR = 1.36, 95% CI: 1.02–1.81, *p*=0.038).

### 3.3. Dose–Response Relationship and Predictive Ability

**Figure 2:**
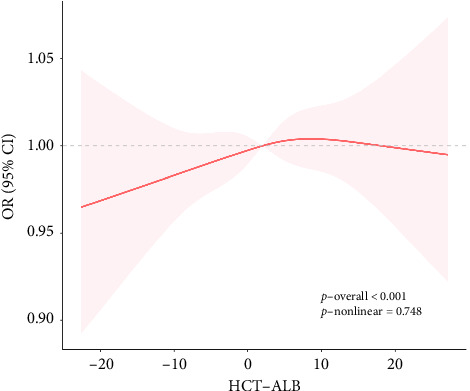
Restricted cubic spline curves between HCT-ALB and the risk of VAP.

The dose–response relationship between HCT-ALB and the risk of VAP was further explored using restricted cubic spline plots (Figure 2). For Model 2, there was a linear dose–response relationship between HCT-ALB and the risk of VAP (*P*_overall_ < 0.001, *P*_non‐linear_=0.748).

**Figure 3:**
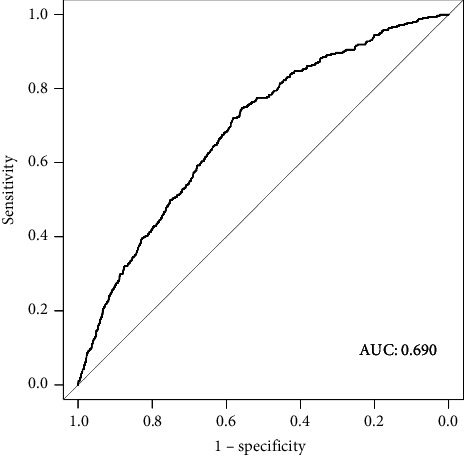
Receiver operator characteristic (ROC) curve for evaluating the predictive ability of HCT-ALB.

**Figure 4:**
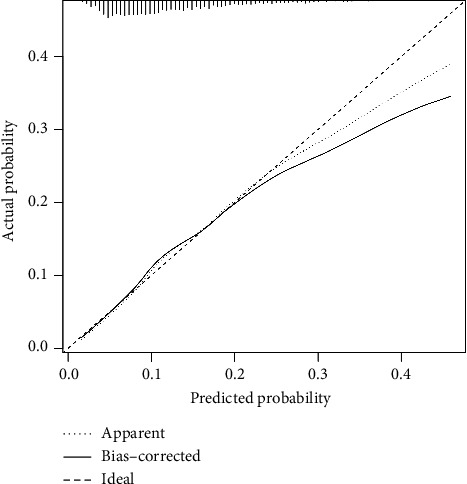
Calibration curve for evaluating the predictive ability of HCT-ALB.

The ROC curve was used to evaluate the predictive efficacy of HCT-ALB on patients with VAP. As shown in Figure 3, the AUC value for Model 2 was 0.690, which demonstrated approximately moderate predictive ability. The calibration curve (Figure 4) further indicated that the model's predicted probabilities were well-aligned with the actual outcomes, suggesting satisfactory calibration performance.

### 3.4. Subgroup Analysis

We performed stratified analyses to evaluate the relationship of HCT-ALB and VAP risk in patients undergoing continuous mechanical ventilation across different subgroups. The results of subgroup analyses based on gender (male/female), qSOFA score (> 2/≤ 2), trauma injury (No/Yes), antibiotics (No/Yes), oral care (No/Yes), COPD (No/Yes), and respiratory failure (No/Yes) were presented in Table 3. To be specific, for patients with continuous mechanical ventilation who were male (OR = 1.51, 95% CI: 1.04–2.19, *p*=0.032), had qSOFA score ≤ 2 (OR = 2.13, 95% CI: 1.35–3.35, *p*=0.001), had not using antibiotics (OR = 1.55, 95% CI: 1.10–2.17, *p*=0.012), had oral care (OR = 1.36, 95% CI: 1.01–1.83, *p*=0.047), and had no history of trauma injury (OR = 1.41, 95% CI: 1.02–1.94, *p*=0.038), COPD (OR = 1.38, 95% CI: 1.02–1.87, *p*=0.036), or respiratory failure (OR = 1.60, 95% CI: 1.08–2.38, *p*=0.021), the association between high HCT-ALB and VAP risk remained statistically significant in the fully adjusted model.

## 4. Discussion

This study revealed that elevated HCT-ALB levels were linked to an increased VAP risk for patients undergoing continuous mechanical ventilation, based on data from a US cohort. The ROC curve demonstrated approximately moderate predictive ability. In addition, our study also revealed the significant relationships between HCT-ALB and VAP in subgroups of male, qSOFA score ≤ 2, not using antibiotics, having oral care, and no history of trauma injury, COPD, or respiratory failure.

The HCT is a hematological parameter that quantifies the proportion of red blood cells to plasma in whole blood, which can be utilized for aiding in the identification of conditions such as anemia or polycythemia [14]. Previous studies have indicated that HCT can serve as a key prognostic biomarker for multiple types of cancer [15, 16], sepsis [17], heart failure [18], and geriatric hip fractures [19]. ALB is a crucial protein that has been demonstrated to exhibit a positive correlation with inflammation severity and disease prognosis [20, 21]. In terms of its physiological mechanism, serum ALB serves various functions in plasma, including extracellular antioxidant activity, buffering capacity, immunomodulation, detoxification, and transportation [22]. Furthermore, the levels of ALB can also be influenced by factors such as a patient's nutritional status or chronic inflammation [23]. Integration of HCT and ALB assessments may be more helpful for the management of patients. It has been shown that the difference between HCT and ALB was regarded as a promising clinical prognostic factor. A study investigated the relationship between HCT-ALB and mortality in elderly patients with sepsis, and the result revealed that high HCT-ALB (≥ 6.7) was related to a 1.41 times higher risk of ICU mortality and a 1.27 times higher risk of hospital mortality among this population [12]. Dai et al. found that HCT-ALB greater than 12.65 could serve as a potential biomarker for the auxiliary diagnosis of preeclampsia and eclampsia in pregnant women with hypertensive disorders [24]. Thus far, there are few studies on the association of the HCT-ALB level with VAP risk among patients with continuous mechanical ventilation. Our study demonstrated a significant association between HCT-ALB ≥ 5.30 and an increased risk of VAP in patients undergoing continuous mechanical ventilation. Importantly, this relationship remained robust even after employing multivariate analysis to control for potential confounding factors. In other words, the susceptibility to VAP may be increased in patients receiving continuous mechanical ventilation with higher HCT values or lower ALB levels. A higher HCT may lead to an increase in blood viscosity and flow resistance [25], thereby potentially impacting respiratory function and predisposing individuals to lung infections [26]. In addition to this, the decrease in serum ALB levels has a direct impact on the functioning of innate immunity and antimicrobial defenses, thereby increasing the susceptibility to infection complications [6]. Ścisło L et al. also showed that VAP occurred more frequently in patients with a lower ALB level [27]. More biological mechanisms underlying the impact of HCT-ALB on VAP risk necessitate further exploration in future studies.

In addition, our study also showed that the high HCT-ALB group and VAP development were significant in some subgroups, such as male, qSOFA score ≤ 2, not using antibiotics, with oral care, without a history of trauma injury, COPD, or respiratory failure. COPD has been a well-recognized risk factor in the development of VAP [28]. Compared to patients without COPD, individuals with COPD exhibit an elevated risk to VAP [29]. For this study, the VAP risk in patients with COPD may surpass the impact of HCT-ALB, which could be one of the reasons why the correlation between HCT-ALB and VAP risk in COPD patients is not statistically significant. However, this explanation could also stem from the limited sample size. From Table 1, we observed that only 25 patients with COPD developed VAP; hence, further studies are warranted to confirm these findings and explore the underlying mechanisms.

To our understanding, this research is the initial attempt to examine the relationship of HCT-ALB with VAP in individuals undergoing continuous mechanical ventilation. HCT-ALB serves as an easily measurable indicator that can assist in risk assessment and facilitate prompt clinical intervention choices. Nevertheless, this study also had some limitations. Firstly, this study relies on retrospective records from the MIMIC-IV database. Despite its rigorous quality control, potential information biases exist. Secondly, incomplete documentation of key variables (e.g., specific protocols for oral care) may compromise the precision of analyzing associations between interventions and VAP. Thirdly, although multiple covariates were included, several potential confounders remain unaddressed (e.g., nutritional status and details of mechanical ventilation). Fourthly, the retrospective design limits clarification of temporal relationships. For example, antibiotic administration was categorized as “used or not,” but differences in timing (e.g., within 24 h preventilation vs. postventilation), duration, and dosage may alter their prophylactic effect on VAP, which could not be further stratified. Lastly, the MIMIC-IV is derived from a single center and lacks diversification; our findings should be interpreted with caution. Further confirmation of our findings necessitates large-scale prospective studies.

## 5. Conclusion

In short, HCT-ALB, as an easily measurable indicator, was associated with a higher risk of VAP in patients with continuous mechanical ventilation.

## Figures and Tables

**Table 1:** Characteristics of all subjects.

Variables	Total (*n* = 3021)	Groups			
		Non-VAP (*n* = 2660)	VAP (*n* = 361)	Statistics	*p*
Age (years), Mean ± SD	61.73 ± 16.54	62.20 ± 16.45	58.29 ± 16.81	*t* = 4.23	< 0.001
Gender, *n* (%)				*χ* ^2^=2.340	0.126
Female	1259 (41.67)	1122 (42.18)	137 (37.95)		
Male	1762 (58.33)	1538 (57.82)	224 (62.05)		
Race/ethnicity, *n* (%)				*χ* ^2^ = 18.646	< 0.001
Black	268 (8.87)	230 (8.65)	38 (10.53)		
White	1879 (62.20)	1690 (63.53)	189 (52.35)		
Other	377 (12.48)	325 (12.22)	52 (14.40)		
Unknown	497 (16.45)	415 (15.60)	82 (22.71)		
Insurance status, *n* (%)				*χ* ^2^ = 8.404	0.004
Medicare	1285 (42.54)	1157 (43.50)	128 (35.46)		
Other	1736 (57.46)	1503 (56.50)	233 (64.54)		
ICU type, *n* (%)				*χ* ^2^ = 9.401	0.094
Neurology	69 (2.28)	59 (2.22)	10 (2.77)		
Cardiology	720 (23.83)	635 (23.87)	85 (23.55)		
Medical	767 (25.39)	659 (24.77)	108 (29.92)		
Surgical	540 (17.87)	484 (18.20)	56 (15.51)		
Medical/Surgical	555 (18.37)	503 (18.91)	52 (14.40)		
Other	370 (12.25)	320 (12.03)	50 (13.85)		
HCT (%), Mean ± SD	31.47 ± 6.78	31.40 ± 6.74	31.98 ± 6.99	*t* = −1.52	0.128
ALB (g/L), Mean ± SD	29.26 ± 6.54	29.32 ± 6.57	28.87 ± 6.33	*t* = 1.22	0.224
HCT-ALB, *n* (%)				*χ* ^2^ = 8.480	0.014
< −1.10	989 (32.74)	893 (33.57)	96 (26.59)		
−1.10–5.30	1026 (33.96)	901 (33.87)	125 (34.63)		
≥ 5.30	1006 (33.30)	866 (32.56)	140 (38.78)		
MAP (mmHg), Mean ± SD	82.38 ± 17.87	82.43 ± 17.77	82.00 ± 18.63	*t* = 0.43	0.669
Heart rate (bpm), Mean ± SD	94.37 ± 22.02	94.49 ± 21.96	93.52 ± 22.47	*t* = 0.79	0.431
Temperature (°C), Mean ± SD	36.67 ± 2.32	36.68 ± 2.24	36.58 ± 2.85	*t* = 0.63	0.531
WBC (K/uL), M (Q_1_, Q_3_)	12.00 (8.30, 17.30)	12.00 (8.30, 17.30)	12.10 (8.10, 17.60)	*Z* = −0.232	0.817
Platelet (K/uL), M (Q_1_, Q_3_)	172.00 (109.00, 252.00)	172.00 (108.00, 254.00)	164.00 (112.00, 247.00)	*Z* = −0.687	0.492
Hemoglobin (g/dL), Mean ± SD	10.31 ± 2.26	10.29 ± 2.24	10.46 ± 2.39	*t* = −1.32	0.186
Creatinine (mg/dL), M (Q_1_, Q_3_)	1.30 (0.80, 2.20)	1.30 (0.80, 2.20)	1.20 (0.80, 2.30)	*Z* = −0.738	0.460
INR (%), M (Q_1_, Q_3_)	1.40 (1.20, 1.90)	1.40 (1.20, 1.90)	1.40 (1.20, 1.80)	*Z* = −1.017	0.309
PT (s), M (Q_1_, Q_3_)	15.30 (13.20, 19.70)	15.30 (13.20, 19.80)	15.20 (13.00, 18.50)	*Z* = −1.900	0.057
Glucose (mg/dL), M (Q_1_, Q_3_)	135.00 (108.00, 180.00)	134.00 (107.50, 179.00)	141.00 (108.00, 182.00)	*Z* = 1.648	0.099
BUN (mg/dL), M (Q_1_, Q_3_)	25.00 (16.00, 44.00)	26.00 (16.00, 44.00)	23.00 (15.00, 43.00)	*Z* = −1.628	0.104
Sodium (mEq/L), Mean ± SD	138.43 ± 5.95	138.29 ± 5.88	139.43 ± 6.38	*t* = −3.20	0.001
Chloride (mEq/L), Mean ± SD	103.73 ± 7.28	103.60 ± 7.23	104.71 ± 7.55	*t* = −2.72	0.006
Bicarbonate (mEq/L), Mean ± SD	22.02 ± 5.50	22.07 ± 5.44	21.62 ± 5.91	*t* = 1.36	0.174
SpO_2_ (%), Mean ± SD	96.21 ± 4.97	96.22 ± 4.88	96.13 ± 5.60	*t* = 0.30	0.767
qSOFA, Mean ± SD	2.32 ± 0.63	2.29 ± 0.63	2.57 ± 0.57	*t* = −8.48	< 0.001
Trauma injury, *n* (%)				*χ* ^2^ = 17.140	< 0.001
No	2606 (86.26)	2320 (87.22)	286 (79.22)		
Yes	415 (13.74)	340 (12.78)	75 (20.78)		
Antibiotics, *n* (%)				*χ* ^2^ = 3.263	0.071
No	2242 (74.21)	1960 (73.68)	282 (78.12)		
Yes	779 (25.79)	700 (26.32)	79 (21.88)		
Oral care, *n* (%)				*χ* ^2^ = 0.144	0.704
No	244 (8.08)	213 (8.01)	31 (8.59)		
Yes	2777 (91.92)	2447 (91.99)	330 (91.41)		
Diabetes, *n* (%)				*χ* ^2^ = 0.158	0.691
No	2132 (70.57)	1874 (70.45)	258 (71.47)		
Yes	889 (29.43)	786 (29.55)	103 (28.53)		
COPD, *n* (%)				*χ* ^2^ = 1.527	0.217
No	2760 (91.36)	2424 (91.13)	336 (93.07)		
Yes	261 (8.64)	236 (8.87)	25 (6.93)		
Respiratory failure, *n* (%)				*χ* ^2^ = 38.273	< 0.001
No	1925 (63.72)	1748 (65.71)	177 (49.03)		
Yes	1096 (36.28)	912 (34.29)	184 (50.97)		

*Note:* HCT-ALB = difference between hematocrit and albumin; SpO_2_ = oxygen saturation; qSOFA = quick Sepsis-related Organ Failure Assessment.

Abbreviations: BUN = blood urea nitrogen, COPD = chronic obstructive pulmonary disease, ICU = intensive care unit, INR = international normalized ratio, MAP = mean arterial pressure, PT = prothrombin time, VAP = ventilator-associated pneumonia, and WBC = white blood cell.

**Table 2:** Relationship between HCT-ALB and the risk of VAP.

Variables	Model 1		Model 2	
	OR (95% CI)	*p*	OR (95% CI)	*p*
HCT-ALB, *n* (%)				
< −1.10	Ref		Ref	
−1.10–5.30	1.29 (0.97–1.71)	0.076	1.25 (0.93–1.67)	0.133
≥ 5.30	1.50 (1.14–1.98)	0.004	1.36 (1.02–1.81)	0.038

*Note:* HCT-ALB = difference between hematocrit and albumin; Model 1: not adjusted confounders. Model 2: adjusting age, race/ethnicity, insurance status, sodium, chloride, quick Sepsis-related Organ Failure Assessment, trauma injury, and respiratory failure.

Abbreviations: CI = confidence interval, OR = odds ratio, and VAP = ventilator-associated pneumonia.

**Table 3:** Subgroup analysis.

Variables	Model 1				Model 2			
	OR (95% CI)	*p*	OR (95% CI)	*p*	OR (95% CI)	*p*	OR (95% CI)	*p*
Subgroup I: Gender	Male		Female		Male		Female	
HCT-ALB, *n* (%)								
< −1.10	Ref		Ref		Ref		Ref	
−1.10–5.30	1.33 (0.91–1.93)	0.137	1.23 (0.80–1.90)	0.341	1.29 (0.88–1.89)	0.198	1.20 (0.76–1.88)	0.431
≥ 5.30	1.65 (1.15–2.37)	0.006	1.25 (0.80–1.94)	0.323	1.51 (1.04–2.19)	0.032	1.10 (0.69–1.75)	0.700

Subgroup II: qSOFA	QSOFA > 2		QSOFA ≤ 2		QSOFA > 2		QSOFA ≤ 2	
HCT-ALB, *n* (%)								
< −1.10	Ref		Ref		Ref		Ref	
−1.10–5.30	1.07 (0.74–1.54)	0.725	1.56 (0.98–2.48)	0.060	1.05 (0.72–1.54)	0.787	1.59 (0.99–2.54)	0.054
≥ 5.30	1.00 (0.69–1.44)	0.988	2.34 (1.50–3.63)	< 0.001	0.99 (0.68–1.46)	0.976	2.13 (1.35–3.35)	0.001

Subgroup III: Trauma injury	No		Yes		No		Yes	
HCT-ALB, *n* (%)								
< −1.10	Ref		Ref		Ref		Ref	
−1.10–5.30	1.27 (0.92–1.75)	0.141	1.41 (0.77–2.60)	0.269	1.24 (0.89–1.72)	0.201	1.33 (0.71–2.52)	0.376
≥ 5.30	1.59 (1.17–2.16)	0.003	1.28 (0.68–2.41)	0.447	1.41 (1.02–1.94)	0.038	1.20 (0.62–2.32)	0.580

Subgroup IV: Antibiotics	No		Yes		No		Yes	
HCT-ALB, *n* (%)								
< −1.10	Ref		Ref		Ref		Ref	
−1.10–5.30	1.45 (1.04–2.03)	0.029	0.96 (0.56–1.62)	0.865	1.42 (1.01–2.01)	0.048	0.86 (0.50–1.49)	0.593
≥ 5.30	1.74 (1.26–2.40)	< 0.001	0.82 (0.45–1.52)	0.537	1.55 (1.10–2.17)	0.012	0.73 (0.38–1.39)	0.334

Subgroup V: Oral care	No		Yes		No		Yes	
HCT-ALB, *n* (%)								
< −1.10	Ref		Ref		Ref		Ref	
−1.10–5.30	1.84 (0.72–4.70)	0.199	1.24 (0.93–1.67)	0.146	1.83 (0.65–5.17)	0.253	1.21 (0.89–1.65)	0.213
≥ 5.30	1.91 (0.71–5.14)	0.200	1.47 (1.11–1.96)	0.008	1.42 (0.47–4.29)	0.535	1.36 (1.01–1.83)	0.047

Subgroup VI: COPD	No		Yes		No		Yes	
HCT-ALB, *n* (%)								
< −1.10	Ref		Ref		Ref		Ref	
−1.10–5.30	1.32 (0.99–1.77)	0.063	0.87 (0.29–2.63)	0.807	1.30 (0.96–1.76)	0.089	0.75 (0.24–2.40)	0.630
≥ 5.30	1.50 (1.12–2.00)	0.006	1.56 (0.59–4.07)	0.368	1.38 (1.02–1.87)	0.036	1.20 (0.43–3.37)	0.727

Subgroup VII: Respiratory failure	No		Yes		No		Yes	
HCT-ALB, *n* (%)								
< −1.10	Ref		Ref		Ref		Ref	
−1.10–5.30	1.32 (0.89–1.97)	0.171	1.11 (0.74–1.67)	0.605	1.26 (0.84–1.90)	0.262	1.22 (0.80–1.86)	0.357
≥ 5.30	1.86 (1.27–2.73)	0.002	1.01 (0.68–1.51)	0.962	1.60 (1.08–2.38)	0.021	1.13 (0.74–1.72)	0.564

*Note:* HCT-ALB = difference between hematocrit and albumin; qSOFA = quick Sepsis-related Organ Failure Assessment. Model 1: not adjusted confounders. Model 2: adjusting age, race/ethnicity, insurance status, sodium, chloride, qSOFA (not adjusted in Subgroup II), trauma injury (not adjusted in Subgroup III), and respiratory failure (not adjusted in Subgroup VII).

Abbreviations: CI = confidence interval, COPD = chronic obstructive pulmonary disease, OR = odds ratio, and VAP = ventilator-associated pneumonia.

## Data Availability

The datasets generated and/or analyze during the current study are available in the MIMIC-IV database, https://mimic.physionet.org/iv/.

## Supporting Information

Supporting Information 1 Supporting Table 1: Sensitivity analysis for before and after data interpolation.

Supporting Information 2 Supporting Table 2: Selection of confounding factors related to VAP.

## References

[1] Wunsch H., Wagner J., Herlim M., Chong D. H., Kramer A. A., Halpern S. D. (2013). ICU Occupancy and Mechanical Ventilator Use in the United States. *Critical Care Medicine*.

[2] Papazian L., Klompas M., Luyt C. E. (2020). Ventilator-Associated Pneumonia in Adults: A Narrative Review. *Intensive Care Medicine*.

[3] Luo W., Xing R., Wang C. (2021). The Effect of Ventilator-Associated Pneumonia on the Prognosis of Intensive Care Unit Patients Within 90 Days and 180 Days. *BMC Infectious Diseases*.

[4] Wu N., Liu T., Tian M. (2023). Albumin, an Interesting and Functionally Diverse Protein, Varies From ‘Native’ to ‘Effective’ (Review). *Molecular Medicine Reports*.

[5] Nour M., Hegazy A., Mosbah A., Abdelaziz A., Fawzy M. (2021). Role of Microalbuminuria and Hypoalbuminemia as Outcome Predictors in Critically Ill Patients. *Critical Care Research and Practice*.

[6] Wiedermann C. J. (2021). Hypoalbuminemia as Surrogate and Culprit of Infections. *International Journal of Molecular Sciences*.

[7] Udzik J., Biskupski A., Szylińska A., Kowalska Z., Listewnik M. (2021). Percentage of Hematocrit Decrease After the Initiation of Cardiopulmonary Bypass-Clinical Implications and Affecting Factors. *Reviews in Cardiovascular Medicine*.

[8] Hao Y., Sun J., Wang X. (2024). Difference in Hematocrit and Plasma Albumin Levels as an Early Biomarker of Severity and Prognosis in Patients With Severe Fever and Thrombocytopenia Syndrome. *Journal of Medical Virology*.

[9] Liang P., Wei Z., Xia J., Yu F. (2024). Correlation of HCT-ALB, SmtO2, CRT and LAC With Renal Impairment and Prognosis in Patients With Septic Shock. *Journal of Medical Biochemistry*.

[10] Liu Q., Lu W., Zhou S., Chen X., Sun P. (2025). A U Shaped Association Between the HCT-ALB and Hospital Mortality in Patients With Sepsis. *Scientific Reports*.

[11] Dai D. M., Wang D., Hu D. (2020). Difference in Hematocrit and Plasma Albumin Levels as an Additional Biomarker in the Diagnosis of Infectious Disease. *Archives of Medical Science*.

[12] Wang Z., Zhang L., Li S. (2022). The Relationship Between Hematocrit and Serum Albumin Levels Difference and Mortality in Elderly Sepsis Patients in Intensive Care Units-A Retrospective Study Based on Two Large Database. *BMC Infectious Diseases*.

[13] Cai W., Xu J., Wu X. (2023). Association Between Triglyceride-Glucose Index and All-Cause Mortality in Critically Ill Patients With Ischemic Stroke: Analysis of the MIMIC-IV Database. *Cardiovascular Diabetology*.

[14] Kiya G. T., Zewudie F. M. (2019). Comparison of Three-fold Converted Hematocrit and Micro-hematocrit in Pregnant Women. *PLoS One*.

[15] Chen J., Li Y., Cui H. (2021). Preoperative Low Hematocrit is an Adverse Prognostic Biomarker in Ovarian Cancer. *Archives of Gynecology and Obstetrics*.

[16] Lin J. X., Lin J. P., Xie J. W. (2019). Preoperative Hematocrit (HCT) is a Novel and Simple Predictive Marker for Gastric Cancer Patients Who Underwent Radical Gastrectomy. *Annals of Surgical Oncology*.

[17] Luo M., Chen Y., Cheng Y., Li N., Qing H. (2022). Association Between Hematocrit and the 30-Day Mortality of Patients With Sepsis: A Retrospective Analysis Based on the Large-Scale Clinical Database MIMIC-IV. *PLoS One*.

[18] Rao J., Ma Y., Long J., Tu Y., Guo Z. (2023). The Combined Impact of Hyponatremia and Hematocrit on the Risk for 90-Day Readmission and Death in Patients With Heart Failure: Dilutional Hyponatremia Versus Depletional Hyponatremia. *Annals of Saudi Medicine*.

[19] Zhang Y. M., Li K., Cao W. W., Chen S. H., Zhang B. F. (2023). The Effect of Hematocrit on All-Cause Mortality in Geriatric Patients With Hip Fractures: A Prospective Cohort Study. *Journal of Clinical Medicine*.

[20] Yang K., Yang N., Sun W. (2023). The Association Between Albumin and Mortality in Patients With Acute Kidney Injury: A Retrospective Observational Study. *BMC Nephrology*.

[21] Wang X., Xu J., Zhang H., Qu P. (2023). The Effect of Albumin and Hemoglobin Levels on the Prognosis of Early-Stage Cervical Cancer: A Prospective, Single-Center-Based Cohort Study. *BMC Women’s Health*.

[22] Artigas A., Wernerman J., Arroyo V., Vincent J. L., Levy M. (2016). Role of Albumin in Diseases Associated With Severe Systemic Inflammation: Pathophysiologic and Clinical Evidence in Sepsis and in Decompensated Cirrhosis. *Journal of Critical Care*.

[23] Nogueira Á., Álvarez G., Barril G. (2022). Impact of the Nutrition-Inflammation Status on the Functionality of Patients With Chronic Kidney Disease. *Nutrients*.

[24] Dai D. M., Cao J., Yang H. M. (2017). Hematocrit and Plasma Albumin Levels Difference may be a Potential Biomarker to Discriminate Preeclampsia and Eclampsia in Patients With Hypertensive Disorders of Pregnancy. *Clinica Chimica Acta*.

[25] Nader E., Skinner S., Romana M. (2019). Blood Rheology: Key Parameters, Impact on Blood Flow, Role in Sickle Cell Disease and Effects of Exercise. *Frontiers in Physiology*.

[26] Sun Y., Zhao T., Li D., Huo J., Hu L., Xu F. (2019). Predictive Value of C-Reactive Protein and the Pediatric Risk of Mortality III Score for Occurrence of Postoperative Ventilator-Associated Pneumonia in Pediatric Patients With Congenital Heart Disease. *Pediatric Investigation*.

[27] Ścisło L., Walewska E., Bodys-Cupak I., Gniadek A., Kózka M. (2022). Nutritional Status Disorders and Selected Risk Factors of Ventilator-Associated Pneumonia (VAP) in Patients Treated in the Intensive Care Ward-A Retrospective Study. *International Journal of Environmental Research and Public Health*.

[28] Kózka M., Sega A., Wojnar-Gruszka K., Tarnawska A., Gniadek A. (2020). Risk Factors of Pneumonia Associated With Mechanical Ventilation. *International Journal of Environmental Research and Public Health*.

[29] Rouzé A., Cottereau A., Nseir S. (2014). Chronic Obstructive Pulmonary Disease and the Risk for Ventilator-Associated Pneumonia. *Current Opinion in Critical Care*.

