# Supplementary material for: Association of Hematocrit and Albumin Difference With Ventilator-Associated Pneumonia in Patients With Continuous Mechanical Ventilation: Evidence From MIMIC-IV Database
Source: Can Respir J. 2025 Nov 6;2025:6084081. doi: 10.1155/carj/6084081 (PMC12615028; doi:10.1155/carj/6084081)
Supplement: Supporting Information 1 — Supporting Table 1: Sensitivity analysis for before and after data interpolation. [file 6084081.f1.docx]

**Supplementary Table 1 Sensitivity analysis for before and after data interpolation**

| **Variables** | **Before interpolation (n=3021)** | **After interpolation (n=3021)** | **Statistics** | ***P*** |
| --- | --- | --- | --- | --- |
| MAP, mmHg, Mean ± SD | 82.37 ± 17.87 | 82.38 ± 17.87 | t=-0.01 | 0.989 |
| Temperature, ℃, Mean ± SD | 36.68 ± 2.27 | 36.67 ± 2.32 | t=0.23 | 0.821 |
| WBC, K/uL, M (Q_1_, Q_3_) | 12.00 (8.30, 17.35) | 12.00 (8.30, 17.30) | Z=0.029 | 0.976 |
| Platelet, K/uL, M (Q_1_, Q_3_) | 172.00 (108.50, 253.00) | 172.00 (109.00, 252.00) | Z=0.015 | 0.988 |
| Hemoglobin, g/dL, Mean ± SD | 10.31 ± 2.26 | 10.31 ± 2.26 | t=0.05 | 0.958 |
| INR, %, M (Q_1_, Q_3_) | 1.40 (1.20, 1.80) | 1.40 (1.20, 1.90) | Z=1.575 | 0.115 |
| PT, seconds, M (Q_1_, Q_3_) | 15.40 (13.20, 19.80) | 15.30 (13.20, 19.70) | Z=0.396 | 0.692 |
| Glucose, mg/dL, M (Q_1_, Q_3_) | 135.00 (108.00, 180.00) | 135.00 (108.00, 180.00) | Z=-0.018 | 0.986 |
| BUN, mg/dL, M (Q_1_, Q_3_) | 25.00 (16.00, 44.00) | 25.00 (16.00, 44.00) | Z=0.018 | 0.986 |
| Sodium, mEq/L, Mean ± SD | 138.43 ± 5.95 | 138.43 ± 5.95 | t=0.01 | 0.994 |
| Bicarbonate, mEq/L, Mean ± SD | 22.02 ± 5.50 | 22.02 ± 5.50 | t=0.02 | 0.983 |
| SpO2, %, Mean ± SD | 96.21 ± 4.97 | 96.21 ± 4.97 | t=-0.01 | 0.993 |

VAP=Ventilator-associated pneumonia; ICU=intensive care unit; HCT-ALB=difference between hematocrit and albumin; MAP=mean arterial pressure; WBC=white blood cell; INR=international normalized ratio; PT=prothrombin time; BUN=blood urea nitrogen; SpO_2_=oxygen saturation; qSOFA=quick Sepsis-related Organ Failure Assessment; COPD=chronic obstructive pulmonary disease.
