# Supplementary material for: Association of Hematocrit and Albumin Difference With Ventilator-Associated Pneumonia in Patients With Continuous Mechanical Ventilation: Evidence From MIMIC-IV Database
Source: Can Respir J. 2025 Nov 6;2025:6084081. doi: 10.1155/carj/6084081 (PMC12615028; doi:10.1155/carj/6084081)
Supplement: Supporting Information 2 — Supporting Table 2: Selection of confounding factors related to VAP. [file 6084081.f2.docx]

**Supplementary Table 2 Selection of confounding factors related to VAP**

| **Variables** | **OR (95%CI)** | ***P*** |
| --- | --- | --- |
| Age | 0.99 (0.98-0.99) | <0.001 |
| Gender |  |  |
| Female | Ref |  |
| Male | 1.19 (0.95-1.50) | 0.126 |
| Race/ ethnicity |  |  |
| Black | Ref |  |
| White | 0.68 (0.47-0.98) | 0.041 |
| Other | 0.97 (0.62-1.52) | 0.889 |
| Unknown | 1.20 (0.79-1.81) | 0.400 |
| Insurance status |  |  |
| Medicare | Ref |  |
| Other | 1.40 (1.11-1.76) | 0.004 |
| ICU type |  |  |
| Neurology | Ref |  |
| Cardiology | 0.79 (0.41-1.69) | 0.513 |
| Medical | 0.97 (0.50-2.06) | 0.925 |
| Surgical | 0.68 (0.34-1.49) | 0.302 |
| Medical/Surgical | 0.61 (0.31-1.33) | 0.183 |
| Other | 0.92 (0.46-2.02) | 0.828 |
| MAP | 1.00 (0.99-1.00) | 0.669 |
| Heart rate | 1.00 (0.99-1.00) | 0.431 |
| Temperature | 0.98 (0.95-1.03) | 0.456 |
| WBC | 0.99 (0.98-1.00) | 0.232 |
| Platelet | 1.00 (1.00-1.00) | 0.262 |
| Hemoglobin | 1.03 (0.98-1.08) | 0.186 |
| Creatinine | 0.98 (0.92-1.05) | 0.656 |
| INR | 0.97 (0.87-1.07) | 0.498 |
| PT | 0.99 (0.98-1.01) | 0.337 |
| Glucose | 1.00 (1.00-1.00) | 0.445 |
| BUN | 1.00 (0.99-1.00) | 0.261 |
| Sodium | 1.03 (1.01-1.05) | <0.001 |
| Chloride | 1.02 (1.01-1.04) | 0.007 |
| Bicarbonate | 0.99 (0.97-1.01) | 0.147 |
| SpO_2_ | 1.00 (0.98-1.02) | 0.742 |
| QSOFA | 2.18 (1.79-2.66) | <0.001 |
| Trauma injury |  |  |
| No | Ref |  |
| Yes | 1.79 (1.35-2.36) | <0.001 |
| Antibiotics |  |  |
| No | Ref |  |
| Yes | 0.78 (0.60-1.02) | 0.071 |
| Oral care |  |  |
| No | Ref |  |
| Yes | 0.93 (0.62-1.37) | 0.704 |
| Diabetes |  |  |
| No | Ref |  |
| Yes | 0.95 (0.75-1.21) | 0.691 |
| COPD |  |  |
| No | Ref |  |
| Yes | 0.76 (0.50-1.17) | 0.218 |
| Respiratory failure |  |  |
| No | Ref |  |
| Yes | 1.99 (1.60-2.49) | <0.001 |

VAP=Ventilator-associated pneumonia; ICU=intensive care unit; MAP=mean arterial pressure; WBC=white blood cell; INR=international normalized ratio; PT=prothrombin time; BUN=blood urea nitrogen; SpO_2_=oxygen saturation; qSOFA=quick Sepsis-related Organ Failure Assessment; COPD=chronic obstructive pulmonary disease.
